# Supplementary material for: Association between Temporal Glycemic Change and Risk of Pancreatic Cancer in Men: A Prospective Cohort Study
Source: Cancers (Basel). 2022 Jul 13;14(14):3403. doi: 10.3390/cancers14143403 (PMC9323305; doi:10.3390/cancers14143403)
Supplement: Supplementary file 1 [file cancers-14-03403-s001.zip › cancers-1793224-supplementary.pdf]

Table S1. Hazard ratios (HRs) for the association between baseline and recent FBG and pancreatic cancer risk in males.

FBG level		P-Ys (case)	HR (95% *CI*)[a]	Adjusted HR (95% *CI*)[b]	Linear HR (95% *CI*)[c]	*P for trend*
**Baseline FBG (mmol/L)**	**NFG (<5.6)**	963655.72 (88)	Ref	Ref		
	**IFG (5.6~)**	325321.29 (30)	1.012 (0.669, 1.531)	0.939 (0.619, 1.426)	1.038 (0.992, 1.087)	0.109
	**DFG (7.0~)**	111871.75 (17)	1.671 (0.994, 2.808)	1.196 (0.672, 2.13)		
**Recent FBG within 2 years (mmol/L)**	**NFG (<5.6)**	107392.07 (35)	Ref	Ref		
	**IFG (5.6~)**	59991.38 (22)	1.130 (0.663, 1.926)	1.078 (0.629, 1.845)	1.048 (0.996, 1.102)	0.070
	**DFG (7.0~)**	28723.29 (17)	1.825 (1.023, 3.259)*	1.389 (0.682, 2.832)		

FBG, fasting blood glucose; P-Ys, person years; HR, hazard ratio; NFG, normal fasting glucose; IFG, impaired fasting glucose; DFG, diabetes fasting glucose; BMI, body mass index. a: Univariate analysis; b: Adjusted for age, education level, frequency of drinking, frequency of smoking, BMI, anti-diabetes drug use; c:Linear effect as per 0.56 mmol/L (10mg/dL) increase in FBG, and adjusted for age, education level, frequency of drinking, frequency of smoking, BMI, anti-diabetes drug use history. *P < 0.05;** P < 0.01.

Table S2. Hazard ratios (HRs) for the association between annual FBG change and pancreatic cancer risk in males with diabetes at baseline.

Annual FBG change		P-Ys (case)	HR (95% *CI*)[a]	Adjusted HR (95% *CI*)[b]
**Value (mmol/L) per year**	**<-0.3**	25581.94 (4)	1.870 (0.501, 6.976)	1.882 (0.499, 7.095)
	**-0.3~0.3**	54338.50 (5)	Ref	Ref
	**0.3~0.6**	11329.64 (1)	1.009 (0.118, 8.637)	0.978 (0.114, 8.401)
	**≥0.6**	9854.56 (2)	2.513 (0.486, 12.983)	2.296 (0.442, 11.917)
**Percent (%) per year**	**<-3**	27222.49 (4)	1.578 (0.423, 5.884)	1.571 (0.417, 5.913)
	**-3~3**	48844.25 (5)	Ref	Ref
	**3~10**	18718.40 (1)	0.549 (0.064, 4.704)	0.512 (0.060, 4.387)
	**≥10**	6319.50 (2)	3.626 (0.701, 18.759)	3.229 (0.620, 16.819)

FBG, fasting blood glucose; P-Ys, person years; HR, hazard ratio; BMI, body mass index. a: Univariate analysis; b: Adjusted for age, education level, smoking history, drinking history, baseline BMI, BMI change, anti-diabetes drug use. *P < 0.05; **P < 0.01.

Table S3. Hazard ratios (HRs) for the association between temporal FBG change and pancreatic cancer risk.

**Temporal FBG change**		**All males**			**Males without diabetes at baseline**		
		**P-Ys (case)**	**HR (95% *CI*)[a]**	**Adjusted HR (95% *CI*)[b]**	**P-Ys (case)**	**HR (95% *CI*)[a]**	**Adjusted HR (95% *CI*)[b]**
**Value (mmol/L)**	**-0.3~0.3**	310140.98 (11)	Ref	Ref	300780.09 (11)	Ref	Ref
	**<-0.3**	274481.32 (26)	2.664 (1.316, 5.391)**	2.432 (1.193, 4.957)*	224504.25 (18)	2.190 (1.034, 4.637)	2.101 (0.992, 4.450)
	**0.3~1**	315356.15 (21)	1.857 (0.895, 3.851)	1.930 (0.930, 4.005)	306616.62 (21)	1.853 (0.893, 3.844)	1.937 (0.933, 4.019)
	**≥1**	284237.88 (35)	3.371 (1.712, 6.639)**	3.610 (1.818, 7.167)**	251210.72 (31)	3.281 (1.649, 6.530)**	3.779 (1.888, 7.561)**
**Percent (%)**	**-5~5**	267901.01 (10)	Ref	Ref	255344.34 (9)	Ref	Ref
	**<-5**	289965.95 (25)	2.304 (1.106, 4.796)*	2.109 (1.008, 4.411)*	241725.98 (18)	2.110 (0.948, 4.696)	2.030 (0.912, 4.519)
	**5~20**	338970.27 (23)	1.799 (0.856, 3.780)	1.866 (0.888, 3.923)	324617.97 (22)	1.904 (0.877, 4.134)	1.982 (0.912, 4.306)
	**≥20**	287379.10 (35)	3.167 (1.568, 6.397)**	3.431 (1.691, 6.963)**	261423.40 (32)	3.378 (1.612, 7.079)**	3.855 (1.832, 8.111)**

FBG, fasting blood glucose; P-Ys, person years; HR, hazard ratio; BMI, body mass index. a: Univariate analysis; b: Adjusted for age, education level, smoking history, drinking history, baseline BMI, BMI change, anti-diabetes drug use, duration of FBG change. *P < 0.05; **P < 0.01.

Table S4. Sensitivity analysis of hazard ratios (HRs) for the association between annual FBG change value and pancreatic cancer risk in males.

	FBG annual change (mmol/L)			
	**-0.05~0.05**	**<-0.05**	**0.05~0.15**	**≥0.15**
		**Excluding coal-mine workers**		
P-Ys (case)	231049.9 (9)	149624.03 (16)	172820.88 (9)	145760.48 (31)
HR (95% CI)[a]	Ref	2.864 (1.265, 6.483)*	1.345 (0.534, 3.388)	5.699 (2.712, 11.974)**
Adjusted HR (95% *CI*)[b]	Ref	2.610 (1.141, 5.967)*	1.266 (0.502, 3.191)	5.048 (2.375, 10.728)**
		**30≤age≤80**		
P-Ys (case)	341609.13 (10)	219645.27 (26)	267924.23 (15)	232763.52 (42)
HR (95% CI)[a]	Ref	4.237 (2.043, 8.789)**	1.918 (0.862, 4.27)	6.436 (3.229, 12.831)**
Adjusted HR (95% *CI*)[b]	Ref	4.058 (1.943, 8.475)**	1.815 (0.815, 4.042)	5.922 (2.949, 11.894)**
		**18.5≤BMI<30 kg/m²**		
P-Ys (case)	353513.67 (9)	226115.5 (24)	273175.34 (13)	223135.65 (38)
HR (95% CI)[a]	Ref	4.407 (2.048, 9.484)**	1.877 (0.802, 4.39)	7.052 (3.409, 14.588)**
Adjusted HR (95% *CI*)[b]	Ref	4.260 (1.966, 9.231)**	1.768 (0.755, 4.138)	6.353 (3.047, 13.246)**
		**Excluding people who developed cancer ≤3 year from study entry**		
P-Ys (case)	384271.18 (10)	248124.99 (24)	298331.01 (15)	252934.73 (40)
HR (95% CI)[a]	Ref	3.949 (1.888, 8.26)**	1.941 (0.872, 4.321)	6.427 (3.213, 12.854)**
Adjusted HR (95% *CI*)[b]	Ref	3.684 (1.748, 7.765)**	1.813 (0.814, 4.039)	5.577 (2.765, 11.248)**
		**Excluding people with inflammation**		
P-Ys (case)	377872.06 (10)	243077.23 (24)	292802.74 (15)	247816.7 (39)
HR (95% CI)[a]	Ref	3.945 (1.886, 8.253)**	1.944 (0.873, 4.327)	6.259 (3.124, 12.542)**
Adjusted HR (95% *CI*)[b]	Ref	3.664 (1.738, 7.724)**	1.815 (0.815, 4.042)	5.428 (2.686, 10.967)**
		**Excluding people who ever used anti-diabetes drug**		
P-Ys (case)	371444.29 (9)	204878.11 (19)	286320.19 (15)	213771.86 (37)
HR (95% CI)[a]	Ref	4.066 (1.839, 8.991)**	2.169 (0.949, 4.957)	7.534 (3.635, 15.616)**
Adjusted HR (95% *CI*)[b]	Ref	3.874 (1.750, 8.573)**	2.024 (0.885, 4.629)	6.615 (3.177, 13.776)**

FBG, fasting blood glucose; P-Ys, person years; HR, hazard ratio; BMI, body mass index. a: Univariate analysis; b: Adjusted for age, education level, smoking history, drinking history, BMI, anti-diabetes drug use, BMI change. *P<0.05;**P < 0.01.

Table S5. Sensitivity analysis of hazard ratios (HRs) for the association between annual FBG change percent and pancreatic cancer risk in males.

	Annual FBG change (%)			
	**-1%~1%**	**<-1%**	**1%~3%**	**≥3%**
		**Excluding coal-mine workers**		
P-Ys (case)	240703.27 (9)	141222.66 (16)	169886.47 (9)	147442.89 (31)
HR (95% CI)[a]	Ref	3.171 (1.401, 7.178)**	1.427 (0.566, 3.594)	5.864 (2.791, 12.319)**
Adjusted HR (95% *CI*)[b]	Ref	2.879 (1.261, 6.575)*	1.361 (0.540, 3.432)	5.170 (2.442, 10.945)**
		**30≤age≤80**		
P-Ys (case)	357963.23 (11)	205812.79 (25)	261805.25 (14)	236360.89 (43)
HR (95% CI)[a]	Ref	4.152 (2.042, 8.441)**	1.747 (0.793, 3.847)	6.164 (3.178, 11.955)**
Adjusted HR (95% *CI*)[b]	Ref	3.895 (1.904, 7.969)**	1.671 (0.758, 3.683)	5.529 (2.836, 10.779)**
		**18.5≤BMI<30 kg/m²**		
P-Ys (case)	367880.7 (10)	213090.94 (23)	265798.31 (12)	229170.22 (39)
HR (95% CI)[a]	Ref	4.212 (2.004, 8.854)**	1.669 (0.721, 3.863)	6.581 (3.284, 13.187)**
Adjusted HR (95% *CI*)[b]	Ref	4.010 (1.896, 8.484)**	1.589 (0.686, 3.679)	5.821 (2.889, 11.729)**
		**Excluding people who developed cancer ≤3 year from study entry**		
P-Ys (case)	400302.51 (11)	233655.46 (23)	290794.1 (14)	258909.84 (41)
HR (95% CI)[a]	Ref	3.821 (1.862, 7.842)**	1.762 (0.800, 3.881)	6.076 (3.122, 11.825)**
Adjusted HR (95% *CI*)[b]	Ref	3.532 (1.709, 7.296)**	1.669 (0.757, 3.677)	5.234 (2.674, 10.242)**
		**Excluding people with inflammation**		
P-Ys (case)	393578.31 (11)	228902.86 (23)	285446.3 (14)	253641.27 (40)
HR (95% CI)[a]	Ref	3.816 (1.859, 7.832)**	1.764 (0.801, 3.886)	5.923 (3.038, 11.547)**
Adjusted HR (95% *CI*)[b]	Ref	3.516 (1.702, 7.266)**	1.670 (0.758, 3.682)	5.113 (2.608, 10.023)**
		**Excluding people who ever used anti-diabetes drug**		
P-Ys (case)	381025.5 (10)	193833.43 (18)	275306.4 (13)	226249.12 (39)
HR (95% CI)[a]	Ref	3.770 (1.739, 8.171)**	1.806 (0.792, 4.120)	6.890 (3.438, 13.807)**
Adjusted HR (95% *CI*)[b]	Ref	3.598 (1.659, 7.804)**	1.707 (0.748, 3.896)	6.014 (2.989, 12.103)**

FBG, fasting blood glucose; P-Ys, person years; HR, hazard ratio; BMI, body mass index. a: Univariate analysis; b: Adjusted for age, education level, smoking history, drinking history, BMI, anti-diabetes drug use, BMI change. *P < 0.05;**P < 0.01.

Table S6. Sensitivity analysis of hazard ratios (HRs) for the association betweenannual FBG change value and pancreatic cancer risk in males without diabetes at baseline.

	Annual FBG change (mmol/L)			
	**-0.05~0.05**	**<-0.05**	**0.05~0.15**	**≥0.15**
		**Excluding coal-mine workers**		
P-Ys (case)	223431.33 (8)	117187.89 (10)	166973.39 (9)	125599.83 (29)
HR (95% CI)[a]	Ref	2.481 (0.979, 6.288)	1.514 (0.584, 3.923)	6.715 (3.069, 14.693)**
Adjusted HR (95% *CI*)[b]	Ref	2.280 (0.898, 5.789)	1.433 (0.552, 3.717)	6.260 (2.838, 13.807)**
		**30≤age≤80**		
P-Ys (case)	330105.49 (9)	171964.28 (19)	258917.62 (15)	201771.6 (38)
HR (95% CI)[a]	Ref	4.238 (1.917, 9.371)**	2.129 (0.932, 4.865)	7.178 (3.47, 14.849)**
Adjusted HR (95% *CI*)[b]	Ref	4.035 (1.824, 8.928)**	2.02 (0.883, 4.619)	6.796 (3.269, 14.131)**
		**18.5≤BMI<30 kg/m²**		
P-Ys (case)	343349.46 (9)	182841.87 (18)	265303.31 (13)	196227.48 (34)
HR (95% CI)[a]	Ref	3.982 (1.788, 8.867)**	1.875 (0.802, 4.387)	6.961 (3.338, 14.519)**
Adjusted HR (95% *CI*)[b]	Ref	3.863 (1.734, 8.609)**	1.761 (0.752, 4.122)	6.306 (3.005, 13.233)**
		**Excluding people who developed cancer ≤3 year from study entry**		
P-Ys (case)	372628.49 (9)	199350.68 (17)	289222.53 (15)	221404.84 (36)
HR (95% CI)[a]	Ref	3.756 (1.674, 8.429)**	2.155 (0.943, 4.924)	7.101 (3.419, 14.745)**
Adjusted HR (95% *CI*)[b]	Ref	3.582 (1.595, 8.045)**	2.019 (0.883, 4.616)	6.420 (3.073, 13.409)**
		**Excluding people with inflammation**		
P-Ys (case)	366496.58 (9)	195783.77 (17)	283921.04 (15)	217118.04 (35)
HR (95% CI)[a]	Ref	3.739 (1.666, 8.391)**	2.158 (0.944, 4.931)	6.881 (3.307, 14.321)**
Adjusted HR (95% *CI*)[b]	Ref	3.570 (1.590, 8.020)**	2.02 (0.883, 4.619)	6.238 (2.980, 13.056)**
		**Excluding people who ever used anti-diabetes drug during follow-up**		
P-Ys (case)	370151.51 (9)	198057.72 (19)	285471.39 (15)	209745.52 (37)
HR (95% CI)[a]	Ref	4.171 (1.886, 9.223)**	2.168 (0.949, 4.954)	7.633 (3.683, 15.821)**
Adjusted HR (95% *CI*)[b]	Ref	3.983 (1.800, 8.813)**	2.023 (0.885, 4.626)	6.685 (3.210, 13.921)**

FBG, fasting blood glucose; P-Ys, person years; HR, hazard ratio; BMI, body mass index. a: Univariate analysis; b: Adjusted for age, education level, smoking history, drinking history, BMI, anti-diabetes drug use, BMI change. *P < 0.05;**P < 0.01.

Table S7. Sensitivity analysis of hazard ratios (HRs) for the association between annual FBG change percent and pancreatic cancer risk in males without diabetes at baseline.

	Annual FBG change (%)			
	**-1%~1%**	**<-1%**	**1%~3%**	**≥3%**
		**Excluding coal-mine workers**		
P-Ys (case)	228969.13 (8)	110954.92 (10)	225197.32 (15)	68071.07 (23)
HR (95% CI)[a]	Ref	2.695 (1.063, 6.832)*	1.927 (0.817, 4.546)	10.209 (4.564, 22.836)**
Adjusted HR (95% *CI*)[b]	Ref	2.506 (0.987, 6.362)	1.837 (0.778, 4.338)	9.557 (4.228, 21.604)**
		**30≤age≤80**		
P-Ys (case)	339965.49 (10)	161663.38 (18)	351753.32 (24)	109376.81 (29)
HR (95% CI)[a]	Ref	3.969 (1.831, 8.602)**	2.335 (1.117, 4.884)*	9.489 (4.622, 19.479)**
Adjusted HR (95% *CI*)[b]	Ref	3.755 (1.732, 8.143)**	2.218 (1.059, 4.643)*	8.860 (4.287, 18.311)**
		**18.5≤BMI<30 kg/m²**		
P-Ys (case)	352075.46 (10)	172879.69 (17)	357125.19 (21)	105641.78 (26)
HR (95% CI)[a]	Ref	3.685 (1.686, 8.053)**	2.092 (0.985, 4.442)	9.284 (4.474, 19.265)**
Adjusted HR (95% *CI*)[b]	Ref	3.575 (1.635, 7.815)**	1.962 (0.923, 4.172)	8.231 (3.935, 17.219)**
		**Excluding people who developed cancer ≤3 year from study entry**		
P-Ys (case)	382086.11 (10)	188462.31 (16)	391246.3 (24)	120811.83 (27)
HR (95% CI)[a]	Ref	3.464 (1.571, 7.638)**	2.369 (1.133, 4.955)*	9.161 (4.432, 18.934)**
Adjusted HR (95% *CI*)[b]	Ref	3.314 (1.502, 7.312)**	2.220 (1.061, 4.648)*	8.169 (3.922, 17.015)**
		**Excluding people with inflammation**		
P-Ys (case)	375750.15 (10)	185109.32 (16)	383989.87 (23)	118470.09 (27)
HR (95% CI)[a]	Ref	3.447 (1.563, 7.600)**	2.272 (1.081, 4.774)*	9.112 (4.408, 18.836)**
Adjusted HR (95% *CI*)[b]	Ref	3.307 (1.499, 7.296)**	2.132 (1.014, 4.484)*	8.186 (3.93, 17.049)**
		**Excluding people who ever used anti-diabetes drug during follow-up**		
P-Ys (case)	379179.89 (10)	187322.65 (18)	383716.46 (24)	113207.13 (28)

HR (95% CI)[a]	Ref	3.866 (1.784, 8.380)**	2.396 (1.146, 5.010)*	10.034 (4.871, 20.669)**
Adjusted HR (95% *CI*)[b]	Ref	3.705 (1.708, 8.036)**	2.228 (1.064, 4.663)*	8.593 (4.147, 17.809)**

FBG, fasting blood glucose; P-Ys, person years; HR, hazard ratio; BMI, body mass index. a: Univariate analysis; b: Adjusted for age, education level, smoking history, drinking history, BMI, anti-diabetes drug use, BMI change. *P < 0.05;**P < 0.01.

Table S8. Sensitivity analysis of hazard ratios (HRs) for the association between CV of FBG and pancreatic cancer risk in males.

	**CV of FBG**			***P** for trend*
	**<5%**	**5%~15%**	**≥15%**	
**Excluding coal-mine workers**				
P-Ys (case)	114938.31 (2)	426782.94 (37)	157537.96 (26)	
HR (95% CI)[a]	Ref	4.769 (1.149, 19.792)*	9.099 (2.159, 38.344)**	
Adjusted HR (95% *CI*)[b]	Ref	8.579 (2.053, 35.838)**	13.609 (3.185, 58.155)**	
Linear HR (95% *CI*)[c]		1.035 (1.017, 1.053)**		<0.001
**30≤age≤80**				
P-Ys (case)	159002.09 (4)	653691.26 (55)	249252.73 (34)	
HR (95% CI)[a]	Ref	3.182 (1.153, 8.784)*	5.163 (1.832, 14.553)**	
Adjusted HR (95% *CI*)[b]	Ref	6.006 (2.161, 16.692)**	7.901 (2.767, 22.558)**	
Linear HR (95% *CI*)[c]		1.025 (1.008, 1.042)**		0.003
**18.5≤BMI<30 kg/m^2**				
P-Ys (case)	172382.77 (4)	671067.88 (51)	232493.44 (29)	
HR (95% CI)[a]	Ref	3.079 (1.112, 8.523)*	5.044 (1.772, 14.355)**	
Adjusted HR (95% *CI*)[b]	Ref	5.585 (2.002, 15.578)**	7.161 (2.481, 20.673)**	
Linear HR (95% *CI*)[c]		1.022 (1.003, 1.041)*		0.024
**Excluding people who developed cancer ≤3 year from study entry**				
P-Ys (case)	187654.36 (4)	731768.82 (52)	264242.65 (33)	
HR (95% CI)[a]	Ref	3.105 (1.122, 8.587)*	5.440 (1.926, 15.362)**	
Adjusted HR (95% *CI*)[b]	Ref	5.544 (1.99, 15.447)**	7.539 (2.632, 21.593)**	
Linear HR (95% *CI*)[c]		1.025 (1.009, 1.042)**		0.003
**Excluding people with inflammation**				
P-Ys (case)	184882.74 (3)	718865.22 (52)	257824.7 (33)	
HR (95% CI)[a]	Ref	4.183 (1.306, 13.397)*	7.380 (2.262, 24.072)**	
Adjusted HR (95% *CI*)[b]	Ref	7.599 (2.358, 24.491)**	10.339 (3.131, 34.136)**	
Linear HR (95% *CI*)[c]		1.025 (1.008, 1.042)**		0.003
**Excluding people who ever used anti-diabetes drug**				
P-Ys (case)	183397.9 (4)	700651.34 (52)	192369.14 (24)	
HR (95% CI)[a]	Ref	3.216 (1.162, 8.894)*	5.414 (1.878, 15.611)**	
Adjusted HR (95% *CI*)[b]	Ref	5.862 (2.103, 16.341)**	7.475 (2.582, 21.639)**	
Linear HR (95% *CI*)[c]		1.025 (1.005, 1.045)*		0.014

FBG, fasting blood glucose; P-Ys, person years; HR, hazard ratio; BMI, body mass index. a: Univariate analysis; b: Adjusted for age, education level, smoking history, drinking history, baseline BMI, BMI change, anti-diabetes drug use, times of FBG tests, duration of FBG tests; c: Linear effect as per 1 increase in FBG CV, and adjusted for age, education level, smoking history, drinking history, baseline BMI, BMI change, anti-diabetes drug use, times of FBG tests, duration of FBG tests; *P < 0.05;**P < 0.01.

Table S9. Sensitivity analysis of hazard ratios (HRs) for the association between range of FBG and pancreatic cancer risk in males.

	**Range of FBG (mmol/L)**			***P** for trend*
	**<0.5**	**0.5~1.5**	**≥1.5**	
**Excluding coal-mine workers**				
P-Ys (case)	100232.96 (4)	362340.21 (34)	236686.05 (27)	
HR (95% CI)[a]	Ref	2.206 (0.782, 6.220)	2.648 (0.926, 7.577)	
Adjusted HR (95% *CI*)[b]	Ref	5.018 (1.761, 14.297)**	7.852 (2.618, 23.548)**	
Linear HR (95% *CI*)[c]		1.098 (1.045, 1.154)**		<0.001
**30≤age≤80**				
P-Ys (case)	135731.54 (10)	546147.79 (48)	380066.74 (35)	
HR (95% CI)[a]	Ref	1.110 (0.561, 2.195)	1.142 (0.565, 2.308)	
Adjusted HR (95% *CI*)[b]	Ref	2.6 (1.298, 5.206)**	3.463 (1.629, 7.362)**	
Linear HR (95% *CI*)[c]		1.074 (1.021, 1.129)**		0.006
**18.5≤BMI<30 kg/m^2**				
P-Ys (case)	150553.71 (10)	568195.76 (44)	357194.62 (30)	

HR (95% CI)[a]	Ref	1.064 (0.535, 2.117)	1.126 (0.549, 2.307)	
Adjusted HR (95% *CI*)[b]	Ref	2.379 (1.179, 4.800)*	3.107 (1.433, 6.733)**	
Linear HR (95% *CI*)[c]		1.070 (1.011, 1.132)*		0.019
**Excluding people who developed cancer ≤3 year from study entry**				
P-Ys (case)	164075.93 (9)	617415.73 (45)	402174.18 (35)	
HR (95% CI)[a]	Ref	1.198 (0.585, 2.453)	1.391 (0.667, 2.898)	
Adjusted HR (95% *CI*)[b]	Ref	2.67 (1.288, 5.534)**	3.766 (1.718, 8.256)**	
Linear HR (95% *CI*)[c]		1.073 (1.021, 1.128)**		0.005
**Excluding people with inflammation**				
P-Ys (case)	161594.19 (9)	606563.89 (46)	393414.59 (33)	
HR (95% CI)[a]	Ref	1.241 (0.607, 2.539)	1.337 (0.638, 2.799)	
Adjusted HR (95% *CI*)[b]	Ref	2.790 (1.348, 5.775)**	3.611 (1.637, 7.964)**	
Linear HR (95% *CI*)[c]		1.073 (1.021, 1.129)**		0.006
**Excluding people who ever used anti-diabetes drug**				
P-Ys (case)	161407.31 (10)	605298.3 (46)	309712.77 (24)	
HR (95% CI)[a]	Ref	1.127 (0.568, 2.237)	1.123 (0.536, 2.354)	
Adjusted HR (95% *CI*)[b]	Ref	2.560 (1.272, 5.151)**	3.395 (1.565, 7.364)**	
Linear HR (95% *CI*)[c]		1.025 (1.005, 1.045)*		0.014

FBG, fasting blood glucose; P-Ys, person years; HR, hazard ratio; BMI, body mass index. a: Univariate analysis; b: Adjusted for age, education level, smoking history, drinking history, baseline BMI, BMI change, anti-diabetes drug use, times of FBG tests, duration of FBG tests; c: Linear effect as per 0.56 mmol/L (10mg/dL) increase in FBG range, and adjusted for age, education level, smoking history, drinking history, baseline BMI, BMI change, anti-diabetes drug use, times of FBG tests, duration of FBG tests; *P < 0.05;**P < 0.01.

Table S10. Sensitivity analysis of hazard ratios (HRs) for the association between CV of FBG and pancreatic cancer risk in males without diabetes at baseline.

	**CV of FBG**			***P** for trend*
	**<5%**	**5%~15%**	**≥15%**	
**Excluding coal-mine workers**				
P-Ys (case)	111257.88 (2)	406659.69 (35)	115274.87 (19)	
HR (95% CI)[a]	Ref	4.612 (1.109, 19.181)*	8.850 (2.061, 38.006)**	
Adjusted HR (95% *CI*)[b]	Ref	8.429 (2.011, 35.320)**	13.685 (3.167, 59.129)**	
Linear HR (95% *CI*)[c]		1.040 (1.020, 1.061)**		<0.001
**30≤age≤80**				
P-Ys (case)	153734.46 (4)	623921.45 (52)	185103.08 (25)	
HR (95% CI)[a]	Ref	3.070 (1.110, 8.489)*	4.971 (1.729, 14.288)**	
Adjusted HR (95% *CI*)[b]	Ref	5.889 (2.113, 16.414)**	7.802 (2.699, 22.548)**	
Linear HR (95% *CI*)[c]		1.030 (1.011, 1.050)**		0.002
**18.5≤BMI<30 kg/m²**				
P-Ys (case)	167601.39 (4)	644492.12 (48)	175628.6 (22)	
HR (95% CI)[a]	Ref	2.942 (1.061, 8.159)*	4.936 (1.701, 14.326)**	
Adjusted HR (95% *CI*)[b]	Ref	5.396 (1.928, 15.103)**	7.149 (2.448, 20.881)**	
Linear HR (95% *CI*)[c]		1.028 (1.007, 1.049)**		0.008
**Excluding people who developed cancer ≤3 year from study entry**				
P-Ys (case)	182265.15 (4)	701440.6 (49)	198900.8 (24)	
HR (95% CI)[a]	Ref	2.981 (1.076, 8.258)*	5.130 (1.780, 14.784)**	
Adjusted HR (95% *CI*)[b]	Ref	5.401 (1.932, 15.098)**	7.425 (2.560, 21.539)**	
Linear HR (95% *CI*)[c]		1.029 (1.011, 1.049)**		0.002
**Excluding people with inflammation**				
P-Ys (case)	179611.3 (3)	689232.86 (49)	194475.27 (24)	
HR (95% CI)[a]	Ref	4.026 (1.254, 12.921)*	6.965 (2.096, 23.141)**	
Adjusted HR (95% *CI*)[b]	Ref	7.437 (2.301, 24.037)**	10.209 (3.057, 34.09)**	
Linear HR (95% *CI*)[c]		1.030 (1.010, 1.049)**		0.003
**Excluding people who ever used anti-diabetes drug during follow-up**				
P-Ys (case)	181761.72 (4)	696149.27 (52)	185515.14 (24)	
HR (95% CI)[a]	Ref	3.212 (1.161, 8.884)*	5.553 (1.926, 16.012)**	
Adjusted HR (95% *CI*)[b]	Ref	5.873 (2.107, 16.373)**	7.768 (2.682, 22.494)**	
Linear HR (95% *CI*)[c]		1.027 (1.008, 1.047)**		0.006

FBG, fasting blood glucose; P-Ys, person years; HR, hazard ratio; BMI, body mass index. a: Univariate analysis; b: Adjusted for age, education level, smoking history, drinking history, baseline BMI, BMI change, anti-diabetes drug use, times of FBG tests, duration of FBG tests; c: Linear effect as per 1 increase in FBG CV, and adjusted for age, education level, smoking history, drinking history, baseline BMI, BMI change, anti-diabetes drug use, times of FBG tests, duration of FBG tests; *P < 0.05;**P < 0.01.

Table S11. Sensitivity analysis of hazard ratios (HRs) for the association between range of FBG and pancreatic cancer risk in males without diabetes at baseline.

	Range of FBG (mmol/L)			*P* for trend
	<0.5	0.5~1.5	≥1.5	
**Excluding coal-mine workers**				
P-Ys (case)	97729.69 (4)	354059.79 (33)	181402.96 (19)	
HR (95% CI)[a]	Ref	2.151 (0.761, 6.075)	2.388 (0.811, 7.03)	
Adjusted HR (95% *CI*)[b]	Ref	5.070 (1.774, 14.49)**	8.559 (2.801, 26.155)**	
Linear HR (95% *CI*)[c]		1.115 (1.042, 1.194)**		0.002
**30≤age≤80**				
P-Ys (case)	132300.14 (10)	533600.2 (46)	296858.66 (25)	
HR (95% CI)[a]	Ref	1.068 (0.539, 2.119)	1.024 (0.491, 2.135)	
Adjusted HR (95% *CI*)[b]	Ref	2.596 (1.291, 5.222)**	3.687 (1.701, 7.992)**	
Linear HR (95% *CI*)[c]		1.087 (1.017, 1.162)*		0.013
**18.5≤BMI<30 kg/m²**				
P-Ys (case)	147413.7 (10)	556798.93 (42)	283509.48 (22)	
HR (95% CI)[a]	Ref	1.019 (0.510, 2.033)	1.020 (0.482, 2.159)	
Adjusted HR (95% *CI*)[b]	Ref	2.338 (1.153, 4.742)*	3.261 (1.475, 7.208)**	
Linear HR (95% *CI*)[c]		1.081 (1.005, 1.162)*		0.036
**Excluding people who developed cancer ≤3 year from study entry**				
P-Ys (case)	160515.56 (9)	604593.24 (43)	317497.74 (25)	
HR (95% CI)[a]	Ref	1.152 (0.561, 2.366)	1.239 (0.577, 2.660)	
Adjusted HR (95% *CI*)[b]	Ref	2.645 (1.270, 5.509)**	3.988 (1.785, 8.910)**	
Linear HR (95% *CI*)[c]		1.084 (1.016, 1.157)*		0.015
**Excluding people with inflammation**				
P-Ys (case)	158136.02 (9)	594034.21 (44)	311149.2 (23)	
HR (95% CI)[a]	Ref	1.197 (0.584, 2.456)	1.163 (0.537, 2.520)	
Adjusted HR (95% *CI*)[b]	Ref	2.780 (1.337, 5.781)**	3.812 (1.691, 8.592)**	
Linear HR (95% *CI*)[c]		1.084 (1.015, 1.159)*		0.017
**Excluding people who ever used anti-diabetes drug during follow-up**				
P-Ys (case)	160229.86 (10)	602267.82 (46)	300928.46 (24)	
HR (95% CI)[a]	Ref	1.126 (0.568, 2.234)	1.146 (0.547, 2.403)	
Adjusted HR (95% *CI*)[b]	Ref	2.591 (1.288, 5.212)**	3.650 (1.678, 7.937)**	
Linear HR (95% *CI*)[c]		1.027 (1.008, 1.047)**		0.006

FBG, fasting blood glucose; P-Ys, person years; HR, hazard ratio; BMI, body mass index. a: Univariate analysis; b: Adjusted for age, education level, smoking history, drinking history, baseline BMI, BMI change, anti-diabetes drug use, times of FBG tests, duration of FBG tests; c: Linear effect as per 0.56 mmol/L (10mg/dL) increase in FBG range, and adjusted for age, education level, smoking history, drinking history, baseline BMI, BMI change, anti-diabetes drug use, times of FBG tests, duration of FBG tests; *P < 0.05;**P < 0.01.

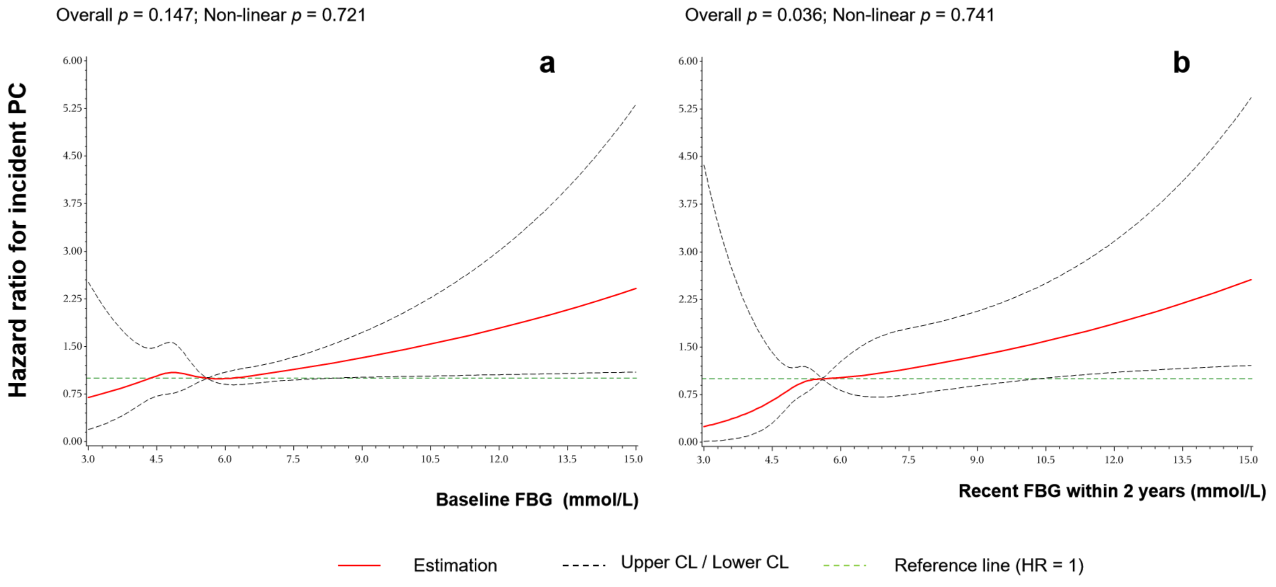

Figure S1. Non-linear associations between FBG levels and pancreatic cancer risk in males.
